# Novel Human Enterovirus C Infection in Child with Community-acquired Pneumonia

**DOI:** 10.3201/eid1811.120321

**Published:** 2012-11

**Authors:** Cristina Daleno, Antonio Piralla, Vytautas Usonis, Alessia Scala, Rimvydas Ivaskevicius, Fausto Baldanti, Nicola Principi, Susanna Esposito

**Affiliations:** Fondazione IRCCS Ca’ Granda Ospedale Maggiore Policlinico, Milan, Italy (C. Daleno, A. Scala, N. Principi, S. Esposito);; Fondazione IRCCS Policlinico San Matteo, Pavia, Italy (A. Piralla, F. Baldanti);; and Vilnius University, Vilnius, Lithuania (V. Usonis, R. Ivaskevicius)

**Keywords:** community-acquired pneumonia, pneumonia, enterovirus, enterovirus C, Picornaviridae, respiratory infection, respiratory virus, viruses, child

**To the Editor:** Human enteroviruses (HEVs) are small, nonenveloped viruses with a positive-stranded RNA genome that includes regions P1, P2, and P3 (*1*). Most experts believe that HEV strains with >75% nt and >85% aa identity in complete or partial viral protein 1 (VP1) sequences should be considered the same type (*2*). However, more stringent criteria of 75% nt and 88% aa identity have been suggested for routine typing (*3*). We report the isolation and characterization of a novel HEV type within the species HEV-C (designated EV-C117 by the *Picornaviridae* Study Group, www.picornastudygroup.com).

A 45-month-old boy was admitted to a hospital in Vilnius, Lithuania, in December 2010 after 1 day of high fever (temperature 40°C) with cough and a moderately increased respiratory rate (36 breaths/min). Decreased breath sounds were heard at the base of the left lung during auscultation, and a chest radiograph showed alveolar pneumonia with partial consolidation of the lower lobe of the left lung. The patient had a leukocyte count of 23,900 cells/mm^3^ and C-reactive protein level of 9.6 mg/dL. Blood culture results were negative for bacteria and fungi. The patient was treated with cefuroxime (500 mg every 8 h) for 7 days. Oxygen administration was not required because the saturation level of peripheral oxygen was always >97%. The patient was discharged in good clinical condition after 7 days and did not experience clinical problems in the following 4 weeks.

For research purposes, a nasopharyngeal sample was collected from the boy at hospital admission by using a flexible pernasal flocked swab; written informed consent was obtained from the parents. The swab was immediately placed in a minitube containing 1 mL of universal transport medium (UTM-RT Kit; Copan Italia, Brescia, Italy). The sample was stored at 4°C in the hospital laboratory before being sent to the central laboratory at the University of Milan, Italy in a refrigerated package. We extracted viral nucleic acids from the swab sample by using an automated extraction system (NucliSens easyMAG; Biomeriéux, Craponne, France), and we tested the extract for respiratory viruses by using the Respiratory Virus Panel ( Fast assay (Luminex Molecular Diagnostics Inc., Toronto, Ontario, Canada) in accordance with the manufacturer’s instructions (*4*).

The assay result was positive for bocavirus and enterovirus/rhinovirus, so we retested the sample to identify the rhinovirus. We performed real-time reverse transcription PCR by using the AgPath-ID One-Step RT-PCR Kit (Applied Biosystems, Foster City, CA, USA) and primers and probe sequences reported by Lu et al. (*5*). Phylogenetic analysis of the VP4/VP2 region showed that some nucleotide sequences belonged to enterovirus species. We obtained a partial VP1 sequence by using the primers described by Nix et al. (*6*), and we obtained the remaining sequence of the VP1 capsid region by using in-house amplification and sequencing protocols (available upon request). The complete P1 sequence was submitted to the *Picornaviridae* Study Group, compared with other enterovirus sequences, and designated as a proposed new type of enterovirus, EV-C117 (GenBank accession no. JQ446368).

To obtain additional viral sequences, we analyzed and sequenced the complete P1 capsid region. This region was compared with the matching region of all of the complete enterovirus genomes available in the GenBank database (as of January 20, 2012). On the basis of the nucleotide sequences, we reconstructed a phylogenetic tree by using maximum likelihood methods with the Tamura 3-parameter model as the evolutionary model; rates among sites were heterogeneous, and gamma distribution was used for the relative rate (*7*). The closest genotypes were EV-C104 (GenBank accession no. EU840733) and EV-C109 (GenBank accession no. NC014336) (Figure). The VP4 genomic region had the greatest identity with other HEV strains; the VP1 coding region had the lowest identity.

**Figure:**
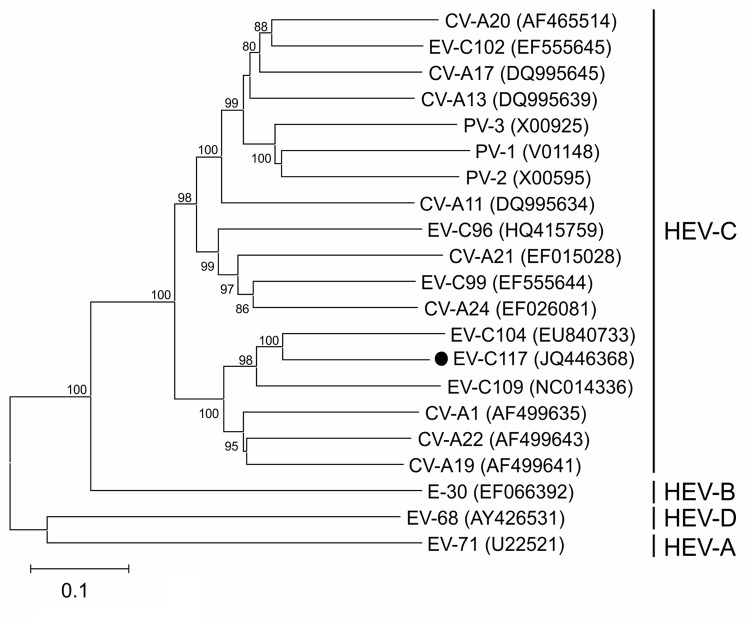
Phylogenetic relationships of human enterovirus C (HEV-C) and the new strain EV-C117 (dot), as determined on the basis of the complete capsid protein coding region sequences. The phylogeny of the nucleotide sequences was reconstructed by using maximum likelihood methods with the Tamura 3-parameter model as the evolutionary model rates among sites were heterogeneous, and gamma distribution was used for the relative rate (*7*). Branch support was assessed by means of bootstrap analyses of 1,000 replicates; a bootstrap value of 70% was used as the cutoff point for cluster analysis. Enterovirus strains EV-68 and EV-70 were used as the outgroup. Scale bar indicates nucleotide substitutions per site.

We report the identification of a novel enterovirus (designated EV-C117) in a child hospitalized with community-acquired pneumonia in Vilnius, Lithuania. EV-C117 was detected in the child in association with bocavirus. Although it is not possible to say whether this new enterovirus was the etiologic cause of the disease, a close relationship has been found (mainly in children) between the development of severe lower respiratory tract infections requiring hospitalization and infections caused by EV-68 (*8*) and EV-C104 and EV-C109 (*9*), which are molecularly similar to EV-C117. In addition, bocavirus is a frequently reported co-pathogen in children with community-acquired pneumonia (*10*). No bacteria or fungi were observed in the blood culture. It is therefore reasonable to think that this new virus may have played a major role in the development of community-acquired pneumonia.

Our findings serve as a reminder that all HEV infections should be closely monitored; knowing the molecular characteristics of virus strains involved in lower respiratory tract infections will help determine appropriate prophylactic and therapeutic measures. However, further studies are needed to determine the tissue tropism and possible pathogenesis of EV-C117 in vivo, and epidemiologic studies are needed to clarify the circulation of this virus strain in countries other than Lithuania.
